# Combining subsidence theory and slope stability analysis method for building damage assessment in mountainous mining subsidence regions

**DOI:** 10.1371/journal.pone.0210021

**Published:** 2019-02-06

**Authors:** Xinpeng Diao, Kan Wu, Dawei Zhou, Jinyun Wang, Zhixin Duan, Zixiang Yu

**Affiliations:** 1 NASG Key Laboratory of Land Environment and Disaster Monitoring, China University of Mining and Technology, Xuzhou, China; 2 School of Environment Science and Spatial Informatics, China University of Mining and Technology, Xuzhou, China; Shandong University of Science and Technology, CHINA

## Abstract

Ground subsidence and surface cracks caused by coal mining are typical man-made geological hazards that can severely damage the ecological environment and buildings. In China, within the theme of sustained and stable development, accurate assessment of mining-related building damage is paramount in order to address the contradiction between coal mining enterprises and building owners. Previous research in China focused mainly on the mining areas of plains, and only a few studies have considered building damage caused by intensive mining in mountainous areas. First, based on field investigation, this study located ground surface cracks and assessed the damage to buildings in the village of Nanyetou in Shanxi Province (China) attributable to the exploitation of the 15110 working face of the Baiyangling coal mine. Second, based on the mining subsidence law and boundary angle, the surface influenced boundary caused by underground mining was determined. However, as the existing subsidence theory cannot adequately explain the phenomenon of building damage, the damage was investigated from the perspective of slope stability analysis, and the slope safety factor before and after working face mining were calculated using the Janbu method. The analytical results showed that slope instability due to a decrease of the safety factor because of the coal mining activity was the principal reason for damage to the village buildings, a finding that was confirmed by field survey and InSAR monitoring displacement. The results of this study could provide guidance and reference for the assessment of building damage caused by underground mining in mountain areas.

## Introduction

Chinese state-owned key coal mines statistical data show that about 8.76 billion tons of coal deposits are located under buildings and about 60% of these are located under village areas [1]. With increasing shortages of coal resources, in order to maintain normal operation, various mining enterprises have been exploiting coal deposits under such village areas. Large-scale exploitation of underground coal resources will inevitably lead to uneven subsidence and displacement of the ground surface, resulting in different degrees of damage to surface buildings. However, many other factors unrelated to mining can also cause damage to buildings, which can lead to disputes between mining enterprises and building owners. Such conflicts are especially intense in areas with more complex natural conditions, where mining operations are conducted near buildings. Therefore, within the theme of sustainable and stable development, accurate technical assessment of mining-related building damage has become necessity.

Much research has been conducted globally on the building damage caused by mining activities. With consideration of the diversity and uncertainty of the factors that cause building damage, Malinowska proposed a fuzzy reasoning method for the assessment of damage to buildings affected by mining [2]. An earlier study by Malinowska and Hejmanowski employed GIS analysis methods to assess the damaging effect of underground mining on buildings in Poland [3]. Saeidi et al. focused on the application and comparison of different methods with a case study; and their research results show that, the Dzegeniuk method [4] is more realistic in comparison of the other empirical methods [5]. The InSAR technique also has been used in assessing the mining‑induced damage to structures in mining subsidence regions [6, 7]. Yang et al. analysed the influence of mining on buildings under various topographic conditions, including areas of plains, hills, and mountains [8]. Other studies have proposed assessment models that utilise and compare various methods for delineating mining damage boundaries [9, 10]. Tan and Deng summarised the typical methods used for the technical assessment of building damage caused by mining exploitation [11]. Similarly, Cui et al. [12] and Xu et al. [13] conducted important research on mining damage assessment methods.

Previous research on mining-related damage produced significant results, but most of the results have been based solely on the prediction of mining subsidence. However, because of the complexities of mining subsidence, a reliable prediction of the mining induced subsidence is still a challenge [14]. The main reason is the existence of numerous interrelated factors, such as the rock masses characteristics, the ground surface topography, the natural precipitation, the method of excavation, which making the subsidence analysis becomes more complex [15]. Furthermore, such building damage assessment work concentrated primarily on the influencing factors, evaluation indicators, and methods of assessing building damage, whereas the effects of mining subsidence-induced secondary disasters have not been considered, for example, the mining-induced reduction of slope stability could lead to slope creep, resulting in building damage. Particularly in mountainous areas, the limitations of conventional subsidence prediction models make it difficult to determine the boundary of mining influence accurately. Previous studies have rarely addressed these aspects.

In this study, we considered a village in Shanxi Province (China) as a case study for conducting research on the technical assessment of mining-related building damage. The fundamental theory of mining subsidence cannot adequately explain the extent of the observed building damage. Therefore, we analysed the effect of slope stability on building damage and obtained conclusions consistent with field survey results. The results revealed the cause of building damage in the mountainous region, and could constitute important reference material for future work on the technical assessment of mining-related damage.

## Study area and field survey

### Study area

The study area (37°20’–37°43’N, 113°20’–114°08’E) is located in Xiyang County of Shanxi Province (China). It lies within the Taihang Mountain range, which has a typical rocky landscape.

The fully mechanised longwall 15110 of the Baiyangling coal mine, which belongs to the Guotou Xiyang Energy Co., Ltd., has an inclined working face of 225 m and a 2420 m panel. The thickness, depth, and inclination of the coal seam are 4.5 m, 350–550 m, and 5°–7°, respectively. After commencing operation in February 2017, it has produced an average daily recovery rate of 3.5 m d^−1^. The mining operations of the 15112 and 15116 longwalls, located within the study area, began in October 2012 and August 2015, respectively; however, during the periods of their operation, no building damage was reported by the local villagers.

**Fig 1:**
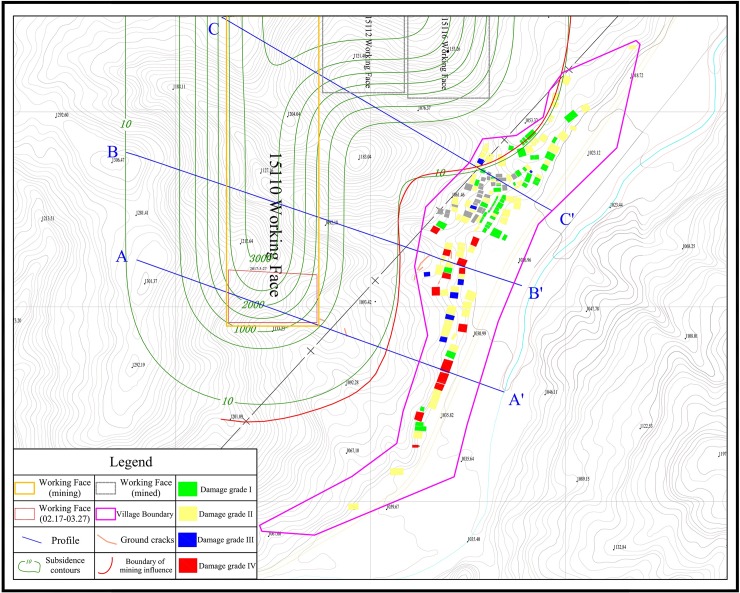
Location of the study area.

**Fig 2:**
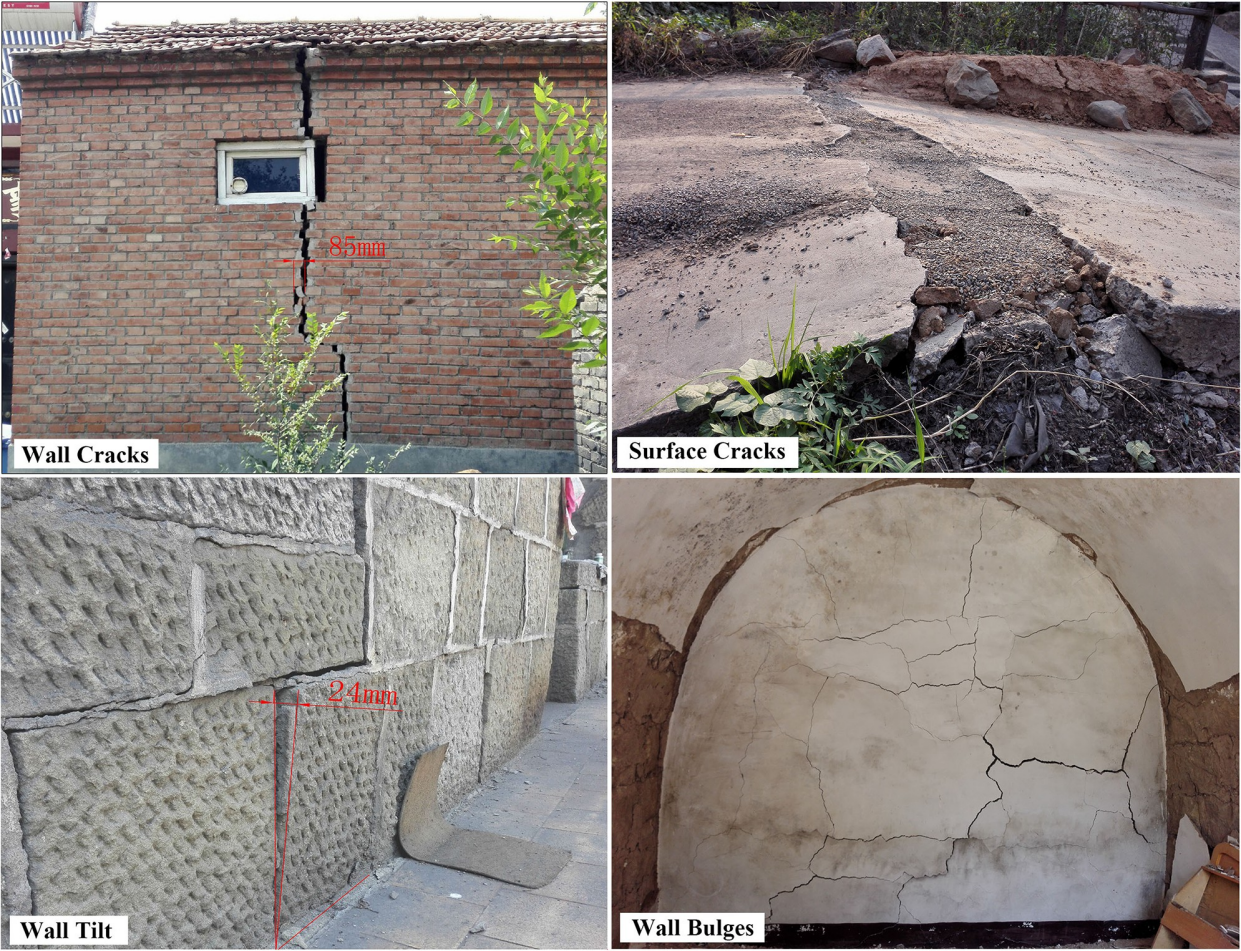
Examples of damage to village buildings. (A) Wall cracks. (B) Surface cracks. (C) Wall tilt. (D) Wall bulges.

The village of Nanyetou is located to the southeast of the Baiyangling coal mine, about 310 m from the boundary of exploitation of longwall 15110. Their relative positions are shown in Fig 1. The buildings in this area mainly consist of step-stone cave homes, single-storey brick and tile structures, and a few brick and mortar houses constructed during the 1960s and 1970s. In March 2017, many villagers reported cracking and tilting of the buildings located on the southern side of the village, as shown in Fig 2.

To facilitate mediation in the dispute between the mine operators and the villagers, a task force began the work of the technical assessment of the damage. The main tasks involved surveying the building damage in the field, followed by grading and assessment of the causes of the damage.

### Field survey methodology

The field survey was divided into two parts: (1) investigation of the extent of building damage and (2) measurement of the surface cracks. The method of investigation of the extent of building damage involved on-site recording of the name(s) of the resident(s) of each building and the details of the damage condition, including the location, shape, number, and width of any cracks and the amount and direction of tilt of any walls. The planimetric locations of surface cracks were established and numbered using GPS, and their widths were measured using a steel tape.

#### Investigation results and analysis

The on-site investigation involved 275 residents and more than 800 buildings. Based on the building damage grading principles of ‘The Regulation for Leaving Coal Pillars and Coal Mining Coal under Buildings, Water Bodies, Railways, and the Main Roadways’ [16], we graded the level of damage of the surveyed buildings (Table 1). Fig 1 shows the distribution of buildings with various grades of damage.

**Table 1:** Cross-reference table of grade of damage to surface buildings and ground deformation.

Damage grade	Description of damaged buildings	Ground deformation value		
		Horizontal deformation (mm m^−1^)	Curvature (10^−3^ m^−1^)	Incline (mm m^−1^)
I	Cracks in walls of masonry buildings <4 mm in width and total width of multiple cracks <10 mm	≤2.0	≤0.2	≤3.0
II	Cracks in walls of masonry buildings <15 mm in width and total width of multiple cracks <30 mm	≤4.0	≤0.4	≤6.0
III	Cracks in walls of masonry buildings <30 mm in width and total width of multiple cracks <50 mm	≤6.0	≤0.6	≤10.0
IV	Cracks in walls of masonry buildings ≥30 mm in width and total width of multiple cracks ≥50 mm	>6.0	>0.6	>10.0

It can be seen from Fig 1 that most of the damage to the buildings on the northern side of the village falls within grades I and II, while on the southern side, further from the boundary of mining influence, some of the damage reaches grades III and IV.

## Analysis of effects of longwall mining

The ground surface displacement caused by underground mining has an impact on the buildings located within the area of influence. Therefore, the primary task in a technical assessment of mining damage is to determine the boundary of influence following longwall mining activity.

### Mining subsidence law and prediction

**Fig 3:**
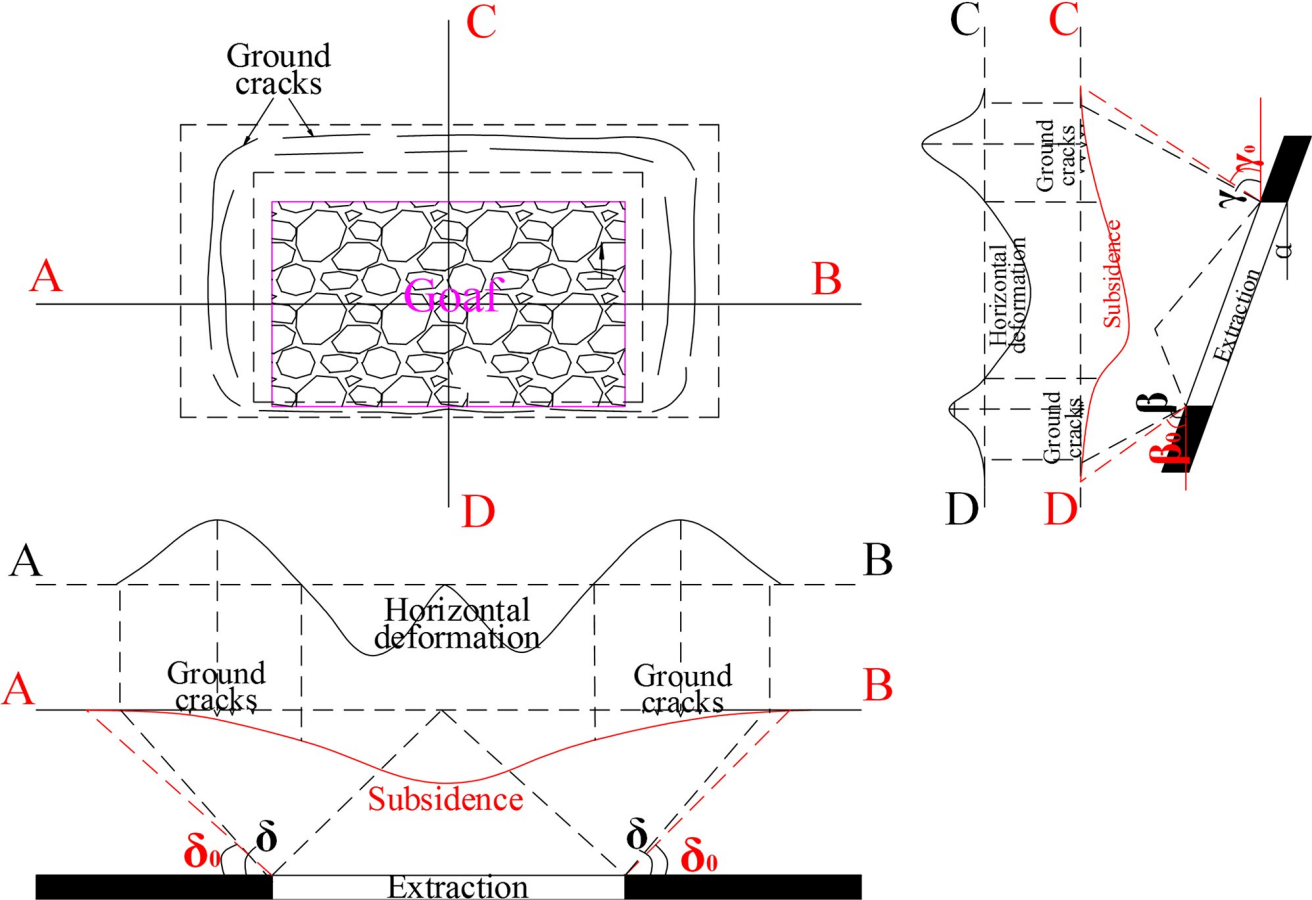
Schematic of ground surface deformation curve and boundary angle.

Because of mining activities, the original balance of rock stress within the study region had changed. The rock strata had been subjected to continuous movement, deformation, and discontinuous damage, resulting in stress redistribution and a new equilibrium [17]. When the area of the underground mining activities reaches a certain extent, the influence of the mining spreads to the ground surface, leading to subsidence, displacement, and deformation, as shown in Fig 3. The range and degree of ground surface deformations are comprehensively affected by various factors, such as mining depth, mining area, mining method, and geological condition [1].

Although the process of mining-related subsidence is complex, the value and spatial distribution of ground surface displacement and deformation have certain regularities: surface subsidence at the top of the exploitation area is the largest, and the value of subsidence from the centre to the edge of the subsidence basin gradually decreases; the spatial distribution of ground surface subsidence is shown by the red curve in Fig 3. Furthermore, ground subsidence and deformation can be calculated using the corresponding method when the underground mining area and geological mining conditions are determined. The stochastic medium model, proposed by Litwiniszyn [18] and generalised by Liu and Liao [19], is one of the most widely used and most well-developed methods. A large number of production practices have demonstrated that the method can achieve a calculation accuracy of 90% relative to the actual deformation observed in mining subsidence areas. Based on this model, in a two-dimensional plane, the surface vertical deformation value *W*_e_(*x*) caused by the mining element can be expressed as follows:

$$W_{e}(x)=\frac{1}{r}e^{-\pi \frac{x^{2}}{r^{2}}}$$ (1)

where *r* = *H*/tan *β* denotes the major influence radius, *H* is the mining depth, and *β* is the mining influence angle; *x* refers to the horizontal coordinate of the mining element.

However, surface deformation is a three-dimensional problem; specifically, the subsidence value at an arbitrary surface point A(*x*, *y*) is the result of the combined effect from two directions: the strike direction (*x* axis) and the tendency direction (*y* axis) of the coal seam. In three dimensions, the final subsidence value *W*_A_(*x*, *y*) can be expressed as

$$W_{A}(x,y)=W_{\max}\iint_{F}f(x,\;y)dF$$ (2)

where *f*(*x*, *y*) is the space probability density function, *F* represents the area of underground coal mining, and *W*_max_ = *mq*cos*α* denotes the maximum subsidence value; *q* is the subsidence coefficient, *m* and *α* represent the average thickness and dip angle of the mined coal seam, respectively.

**Fig 4:**
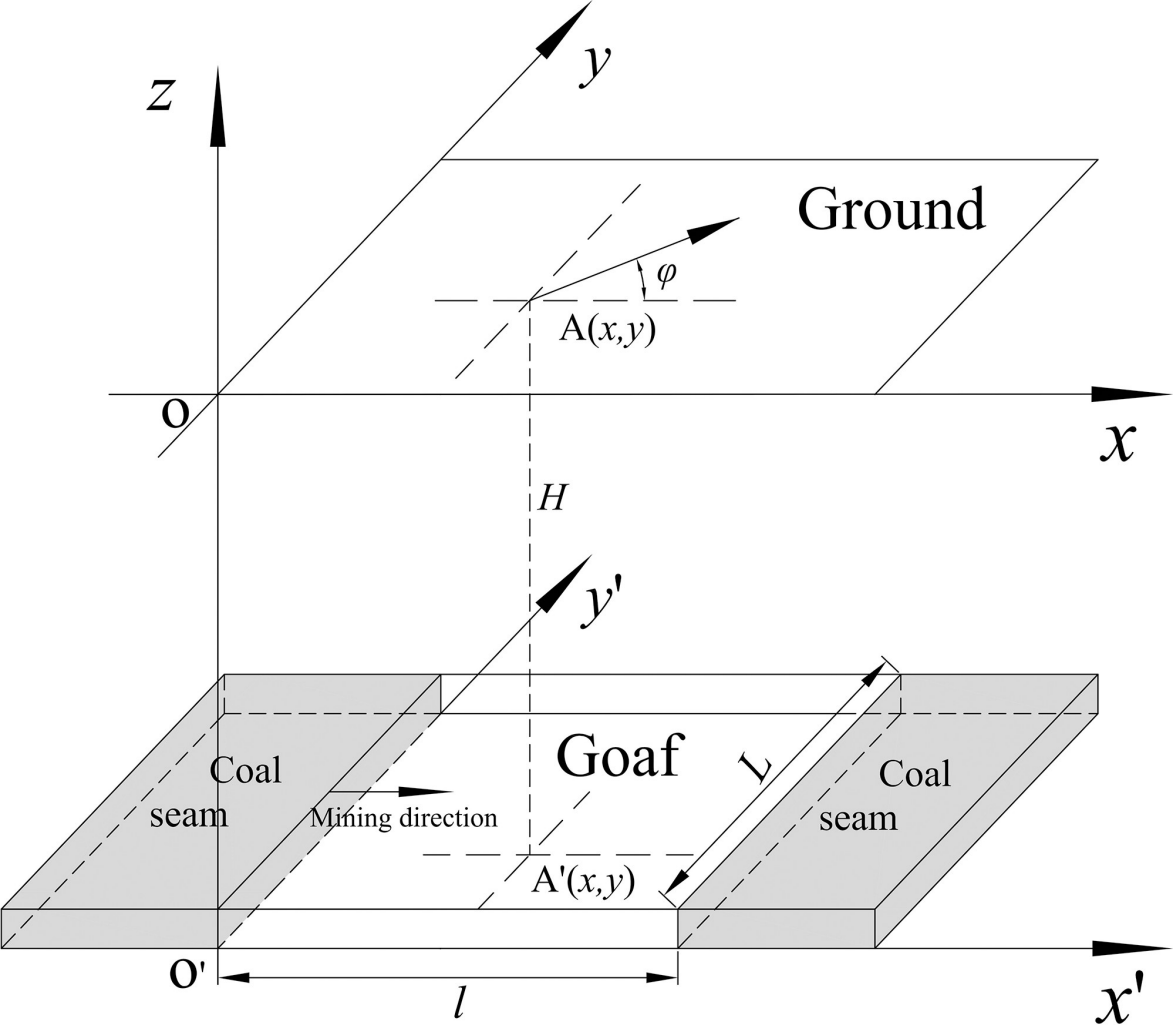
3D coordinate system based on the stochastic medium model.

As shown in Fig 4, *φ* is the included angle between the calculated direction and the positive direction of the *x*-axis in the anticlockwise direction. Assuming that the strike and tendency mining lengths of the rectangular working face are *l* and *L*, respectively, the length of the mined portion of the working face are *x*∈[*0*,*l*] and *y*∈[*0*,*L*]. Taking into account the independence of the probability in the *x* and *y* directions, and taking the lower-left corner of the working face as the origin of the coordinate system, Eq (2) can be expressed as

$$W_{A}(x,\;y)=W_{\max}\iint_{F}f(x)f(y)dxdy=\frac{W_{\max}}{r_{0}{}^{2}}\int_{-x}^{l-x}e^{-\pi {\left(\frac{x}{r_{0}}\right)}^{2}}dx\int_{-y}^{L-y}e^{-\pi {\left(\frac{y}{r_{1(2)}}\right)}^{2}}dy$$ (3)

where *r*_0_ is the major influence radius along the strike direction of the coal seam, and *r*_1(2)_ is the major influence radius along the tendency of the coal seam in the upward (downward) direction.

Eq (3) is the expression for the final subsidence value at an arbitrary surface point induced by underground coal mining. Based on these theories, members of our project team developed software for subsidence predictions based on the AutoCAD platform [20].

In this study, ground subsidence was calculated using the stochastic medium model, for which the predicted parameters (mining thickness: 4500 mm, subsidence coefficient: 0.83, tangent of main influence: 2.10, greatest subsidence angle: 90°, and displacement factor: 0.22) were derived from ‘The Regulation for Leaving Coal Pillars and Coal Mining Coal under Buildings, Water Bodies, Railways, and the Main Roadways’. The calculated contour lines of surface subsidence and the most external influence boundary (the surface subsidence 10mm contour line) are shown by the green line in Fig 1.

The calculated results showed that obvious subsidence occurred on the ground surface because of the mining of underground working surfaces, and that the maximum subsidence reached 3700 mm. The calculated results also indicated theoretically that the outer boundary affected by underground mining would not spread to the village of Nanyetou. However, it is known that the conventional method for the prediction of mining subsidence converges too quickly above the boundary, which makes the resulting range of influence becoming small [11]. It also has limitations in the prediction of subsidence in mountainous areas. Therefore, we used the boundary angle calculation method to determine the boundary of mining influence.

### Determination of longwall mining influence boundary

Assuming that accurate geological data are obtainable, the boundary of longwall mining influence also can be determined based on the boundary angle [1, 17]. The boundary angle is the angle between a line drawn from a border point of the surface movement basin bedding plane to the border of extraction and a horizontal line at the coal pillar side, under the condition of full subsidence or almost full subsidence [17], as shown in Fig 3.

In accordance with the surface movement parameter table in ‘The Regulation for Leaving Coal Pillars and Coal Mining Coal under Buildings, Water Bodies, Railways, and the Main Roadways’, we selected 65° as the boundary angle for the study area [16]. Based on the mining elevations and topography in different locations of longwall 15110 and of previously exploited longwalls 15112 and 15116, we calculated the location of the boundary of mining influence, shown by the red line in Fig 1.

### Analysis and discussion

It can be inferred from Fig 1 that the boundary of mining influence, determined based on the boundary angle, affected only some of the buildings and facilities located in the northern part of the village area. In theory, this means there could be no possibility of damage resulting directly from mining activities to buildings located on the southern side of the village. This contradicts the findings of the on-site investigation.

**Fig 5:**
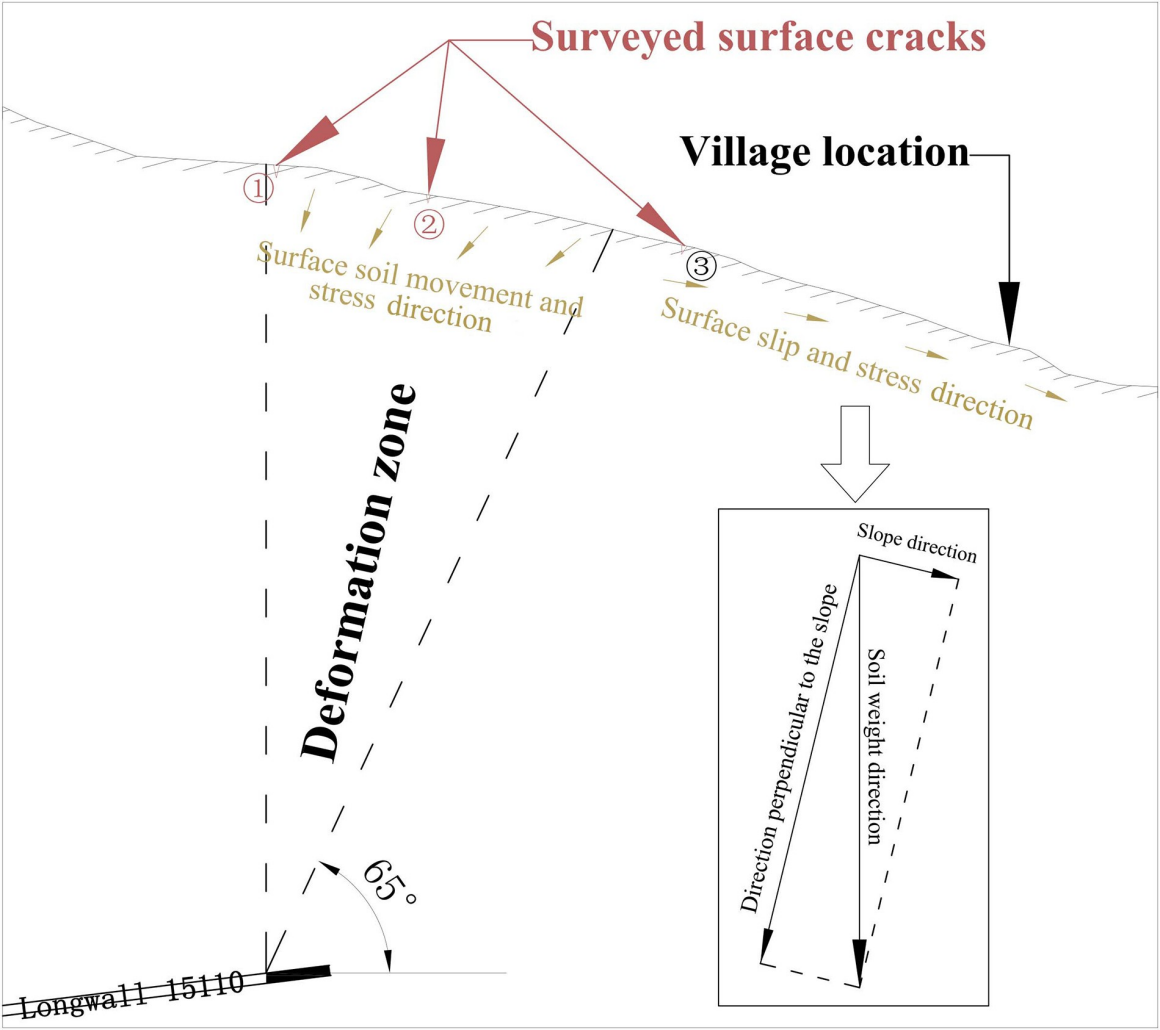
Diagram of surface soil layer stress direction.

The study village is located at the foot of a slope in a mountainous area that has a 5-m-thick surface layer of yellowish-brown clay on top of the bedrock. The stress direction of the surface soil layer in the area after exploitation is shown Fig 5.

Based on the field survey results, it was determined that surface cracks emerged following the longwall mining activity. Fig 5 shows that surface cracks ① and ② are located in the mining-induced tensile zone, where unevenly distributed movement of the surface soil in the direction of the goaf led to the emergence of surface cracks. Surface crack ③ is located outside the boundary of influence. Taking into account the stress direction analysis (Fig 5), it was considered that this crack was caused by the downward force of the soil moving under its own weight along the bedrock. Therefore, the two types of surface cracks have different causes and different characteristics.

Based on the differences in the locations of the surface cracks and on the analysis of their development, we can infer that the damage to the buildings on the southern side of the village was related to the reduction in slope stability due to the emergence of mining-related cracks, which caused slow slippage.

## Slope stability analysis

The slope safety factor is used as the basis for evaluating slope stability. Based on the “Technical Code for Building Slope Engineering”, the slope stability conditions are divided into four categories: stable, mostly stable, slightly unstable, and unstable [21]. The corresponding relationship between the slope condition and the factor of slope safety is shown in Table 2.

**Table 2:** Slope stability condition categories.

Factor of safety	F < 1.00	1.00 ≤ F < 1.05	1.05 ≤ F < F_st_	F ≥ F_st_
**Slope stability condition**	unstable	slightly unstable	mostly stable	stable

### Slope stability analysis methods

Slope stability is a core issue in geotechnical engineering. However, following nearly a century of development, theoretical research has gradually improved understanding of this issue. At present, the quantitative methods available for slope stability analysis include limit equilibrium, reliability analysis, and numerical simulation analysis [22–24]. Among these, the limit equilibrium method, which uses a factor of safety to evaluate slope stability quantitatively, represents the conventional approach used widely in engineering. The most commonly used limit equilibrium methods include the Fellenius method [25], Bishop method [26], Janbu method [27], Sarma method [28], and imbalanced thrust force method [29]. The Janbu method, which is able to satisfy all of the stress equilibrium conditions and to simulate any slip surface shape, is the method most widely used. Therefore, the Janbu method was used in this study to calculate slope stability [30, 31].

**Fig 6:**
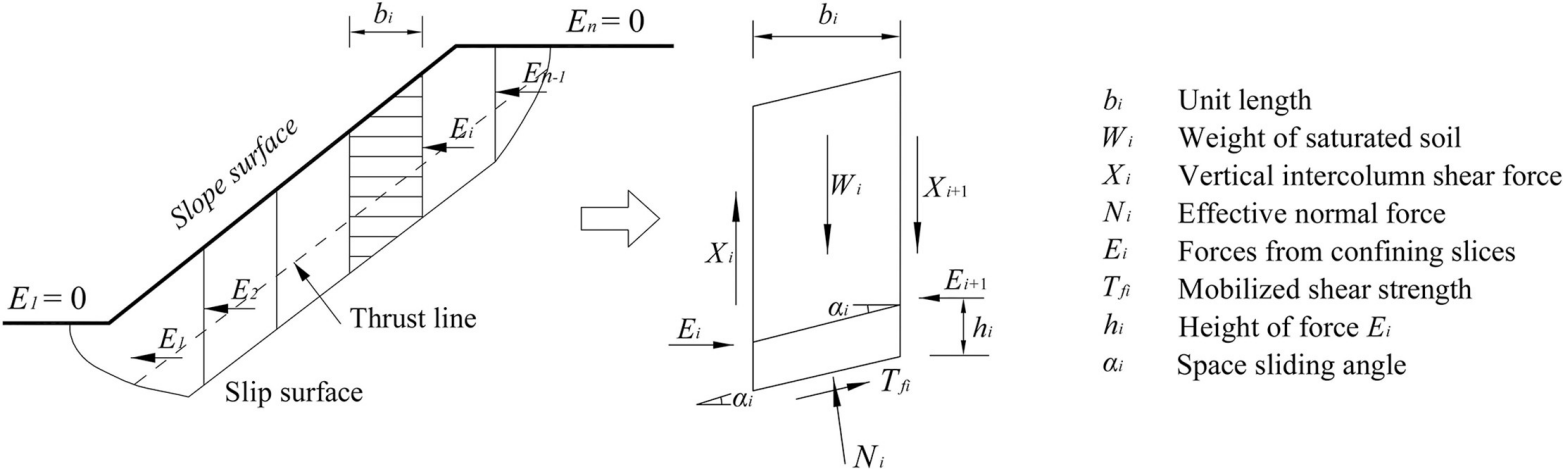
Basic principles of the Janbu slice method.

The basic principles of the Janbu method are as shown in Fig 6. For any arbitrary soil slice *i* within any slip surface, the following is assumed: the tangential force *T*_*i*_ on the slip surface is equal to the shear strength *τ*_*fi*_ of the soil on the slip surface; and the point of application of the bilateral normal force *E* of the soil slip is located at a height of 1/3 above the soil slice.

Based on the vertical equilibrium conditions of a single slice, the following can be obtained:

$$N_{i}cos\alpha_{i}=W_{i}+\Delta X_{i}-T_{fi}sin\alpha_{i}.$$ (4)

Further, based on the horizontal static equilibrium conditions of a single slice, the following can be obtained:

$$\Delta E_{i}=(W_{i}+\Delta X_{i})tan\alpha_{i}-T_{fi}sec\alpha_{i}.$$ (5)

The moment equilibrium at the slice action point can be obtained, while ignoring the higher-order terms:

$$X_{i}={-E}_{i}\;tan\alpha_{ti}+h_{ti}\;\Delta E_{i}/b_{i}.$$ (6)

At the same time, for the entire slope $\sum_{1}^{n}E_{i}=0$, we can obtain the following:

$$\sum_{1}^{n}(W_{i}+\Delta X_{i})\;tan\alpha_{i}-\sum_{1}^{n}T_{fi}sec\alpha_{i}=0.$$ (7)

Based on the definition of safety factor and the Mohr–Coulomb failure criterion, we have:

$$T_{fi}=\left(c_{i}b_{i}+(W_{i}+\Delta X_{i})\;tan\varphi_{i}\right)/\left(F_{s}m_{a_{i}}\right),$$ (8)

where $m_{a_{i}}=cos\alpha_{i}(1+tan\varphi_{i}tan\alpha_{i}/F_{s})$.

After combining Eqs (7) and (8), we obtain the following:

$$F_{s}=\sum \frac{c_{i}b_{i}+(W_{i}+\Delta X_{i})tan\varphi_{i}}{m_{a_{i}}}/\sum (W_{i}+\Delta X_{i})sin\alpha_{i}.$$ (9)

Eq (9) is the slope stability equation of the Janbu method.

### Calculation of original slope safety factor

**Fig 7:**
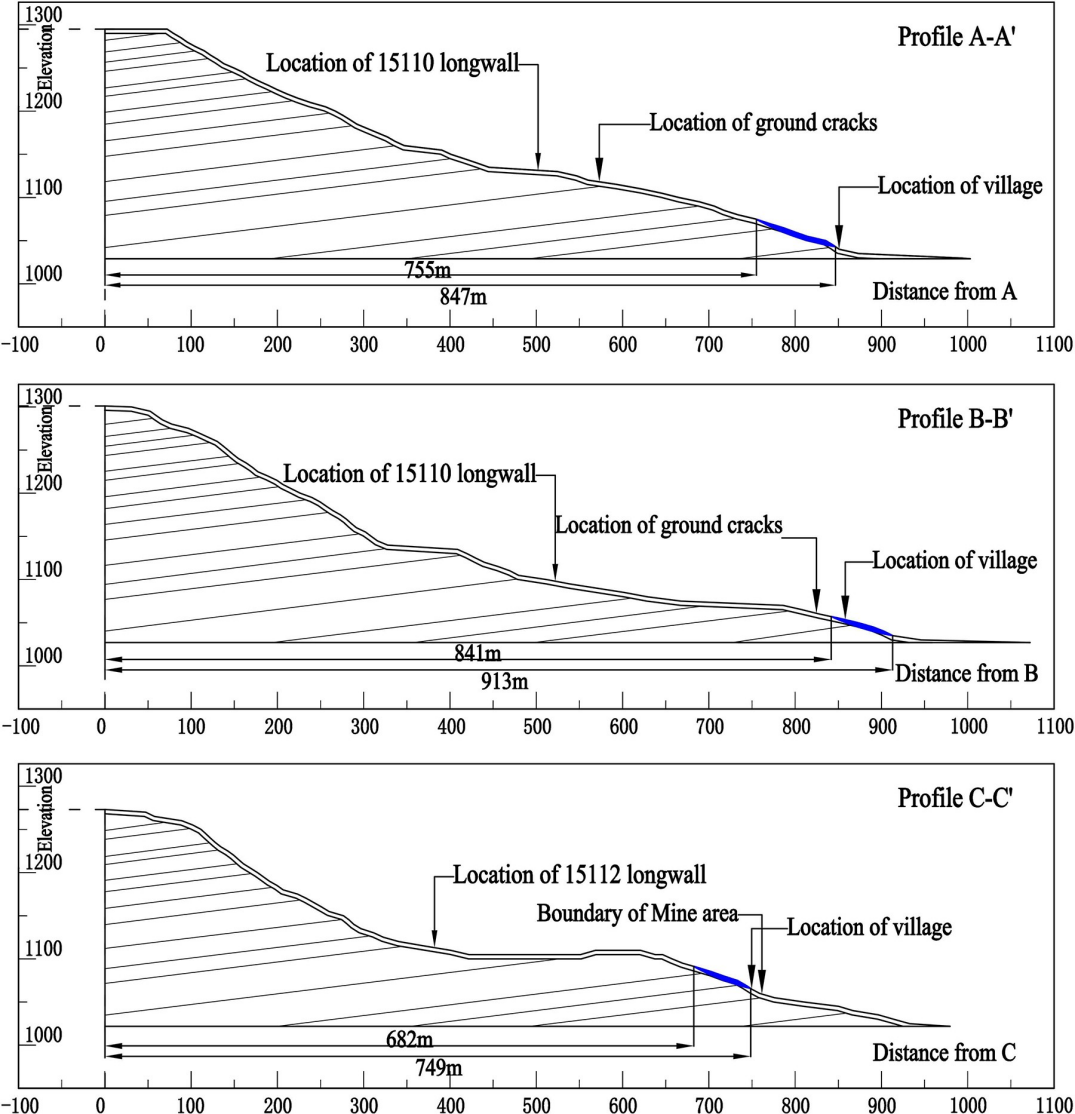
Diagrams of critical slip surface locations at each cross section.

In order to analyse the influence of mining on slope stability, we selected lines A–A', B–B', and C–C' (Fig 1) as the original terrain sections based on the building damage conditions in different areas of the village. Using the equations in the previous section, we calculated the safety factor of original slope (Table 3). The critical slip surface locations at each cross section are shown in Fig 7. The critical slip surface locations of A–A', B–B', and C–C', as measured from points A, B, and C, are at distances of 755–847, 841–913, and 682–749 m, respectively.

**Table 3:** The safety factor of original slope.

**Cross section**	A–A'	B–B'	C–C'
**Factor of safety**	1.169	1.168	1.110

It is evident from Fig 7 that the critical slip surface of the original surface slope is located very close to or is within the village area. The calculated surface slope safety factor presented in Table 3 show that all slopes, without the influence of mining, are categorised as either stable or mostly stable and that the ground structures are not damaged because of slope slip.

### Influence of longwall mining on slope stability

#### Mining-induced slope instability analysis

The main impacts that mining has on slope stability include changes in slope, cracking, and other similar types of damage. When cracks reach certain depths, rainwater seepage can fill them up, which intensifies crack propagation further and reduces slope stability. Previous studies have shown that the slope safety factor decreases with crack expansion and the increase of the depth of the water filling the crack.

The field survey results of this study identified the occurrence of mining-induced cracking near the village, with specific locations shown in Fig 1. Besides, in some of the cave homes located close to the slope side, water seepage through the walls was prominent. Therefore, the slope safety factor would be expected to decrease because of the emergence of surface cracks, and rainwater would inevitably seep into the cracks and penetrate the slope. The impact of rainwater penetration is considered in relation to the following two aspects: ① In the zone of mining-induced slope surface deformation, rainwater infiltration leads to a decrease of the strength parameters of the potential failure surface. Assuming a 10% reduction of soil parameters and unchanged bedrock parameters, we calculated the slope safety factor following longwall mining activity. ② The slope safety factor decreases when the cracks are full of rainwater. Based on the field survey, we used a single crack (depth: 3 m, width: 0.1 m) in the simulation to calculate the slope safety factor following longwall mining activity.

#### Calculation of the slope safety factor following longwall mining activity

Using the Janbu method described in the previous section, we calculated the decrease in the strength parameters of a failure surface caused by rainwater and the change in the slope safety factor following crack water infiltration (Table 4).

**Table 4:** Change in slope safety factor following longwall mining activity.

Cross section	Slope safety factor		
	Original	Following mining activity	Change
A–A'	1.169	0.986	15.7%↓
B–B'	1.168	1.049	10.1%↓
C–C'	1.110	1.085	1.4%↓

It is evident from Table 4 that mining-induced surface cracks caused a decrease in the safety factor of the original slope. The decrease in the safety factor of the critical slip surface of the A–A', B–B', and C–C' cross sections was about 16%, 10%, and 2%, respectively; after the change, the slope conditions were categorised as unstable, slightly unstable, and mostly stable, respectively.

Based on the obtained results, it is determined that a risk of instability exists in the critical slip surface of the A–A' and B–B' cross sections, where landslides due to creep-induced crack instability could occur.

### Analysis and discussion

It is evident from Fig 7 that the critical slip surface of the A–A' cross section is located on the western side of the village boundary. The slip surface in this area presses on the local buildings causing exterior tiles or even entire walls of some of the buildings to tilt. Buildings located within courtyards and close to the slope side were found most affected. The critical slip surface of the B–B' cross section is located within the village. Non-uniform slope slippage causes some ground surface cracking, which leads to cracking of some buildings. The above analysis is consistent with the actual results of the field survey.

## Analysis of displacements monitored by InSAR

**Fig 8:**
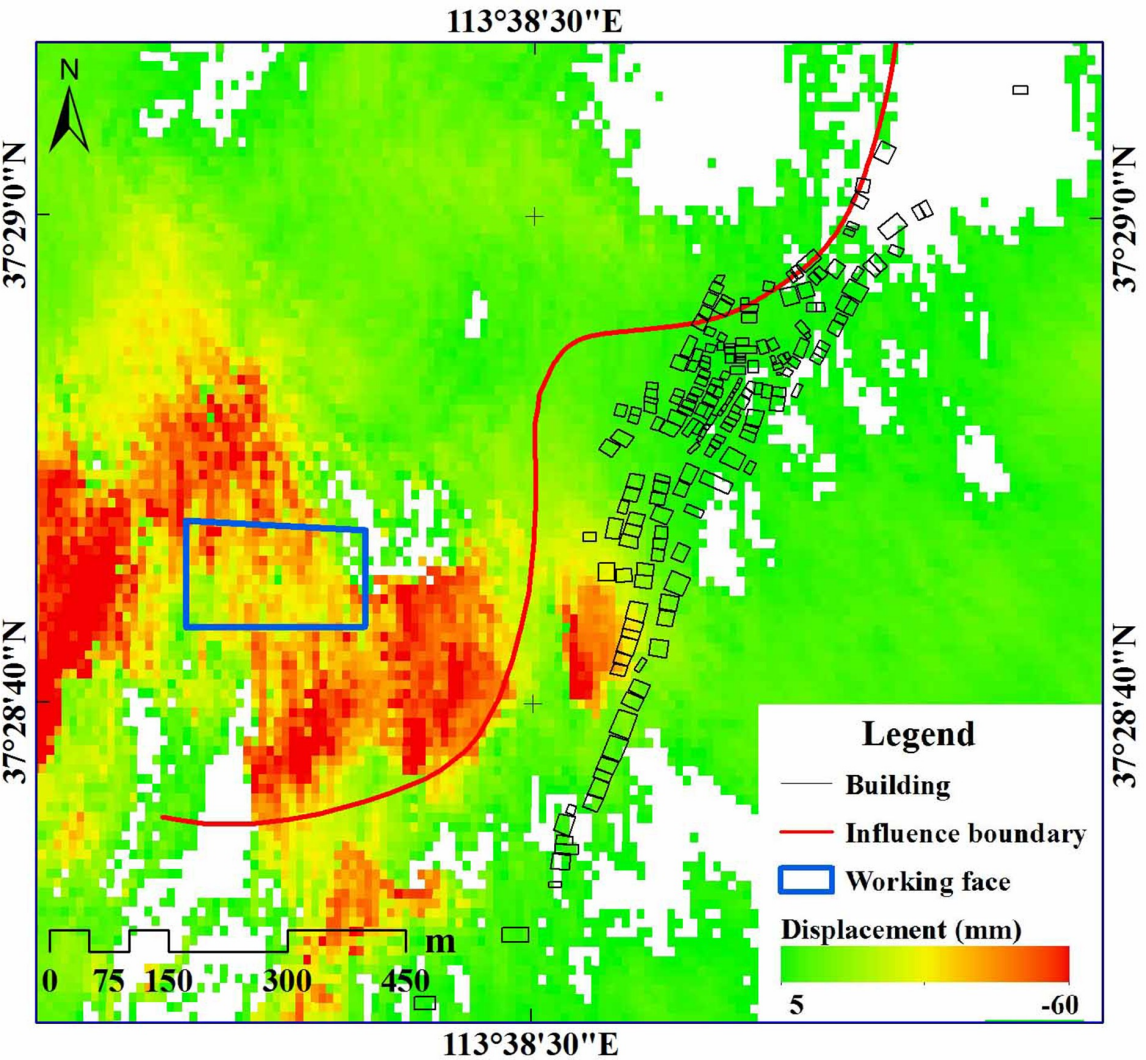
Monitored ground surface displacement using the conventional D-InSAR technique.

In order to verify the results of the slope stability analysis, the conventional D-InSAR technique was used to monitor the surface deformation during the 15110 working face mining (February 17 to March 27, 2017), the mining area are shown by the red line in Fig 1. The SAR data used in this analysis comprised Sentinel-1A images acquired on February 11 and March 31, 2017. The deformation monitoring results are shown in Fig 8.

According to the monitored ground surface displacement results, the following two features were confirmed. ① Mining of the 15110 working face caused obvious ground deformation, and the range of subsidence monitored by the InSAR technique was in good agreement with the mining influence boundary defined in section 3.1. ② Outside the mining influence boundary, near the middle and southern parts of the village, obvious deformation phenomena on the surface validated the slope instability analysed in the previous section.

## Discussion and conclusions

### Discussion

#### Northern area building damage factor analysis

Some of the buildings on the northern side of the village are located within the boundaries of influence of longwalls 15112 and 15116. The influence boundaries were determined using the boundary angle calculation method. The calculation results showed that the largest surface deformation in this area was <4 mm m^−1^. This means that local buildings could have incurred some mining-related damage, but theoretically, the extent of the damage should not reach grade III. Conversely, the slope stability calculation results showed that exploitation of the two longwalls should not have caused any change in the slope stability of this area; thus, mining activity should not have increased the level of damage caused to the buildings. Consequently, the buildings in this area would be expected to be influenced directly only by the longwall mining and their damage grade should be I–II.

The field survey confirmed the existence of only minor cracks in the buildings located in the northern part of the village, consistent with the above analysis.

#### Southern area building damage factor analysis

The analysis in 3th section showed that the southern side of the village is not located within the boundary of influence of longwall 15110, as determined using the boundary angle calculation method. Therefore, under normal topographic conditions, this area would not be influenced directly by the exploitation and the buildings should not show any serious damage. This is contradictory to the results of the field survey. However, the analysis in 4th section indicated that surface cracking induced by longwall mining leads to a decrease in the slope safety factor. Therefore, the change in the slope stability condition is the principal reason for the damage caused to the buildings on the southern side of the village. The building damage in this area was discovered in March 2017, which coincides with the exploitation of longwall 15110 (February 2017). Therefore, although the buildings in this area were not affected directly by the longwall mining, the decrease in slope stability due to the mining activity caused slope creep, which was the direct cause of building damage.

#### Research significance and limitations

This study focused on technical assessment of mining damage based on two aspects: mining subsidence theory and slope stability analysis. As the basic theory of mining subsidence cannot adequately explain the degree of damage to ground structures, this study investigated and analysed the reasons for damage caused to buildings in a village in a mountainous mining area. The results of the analysis were consistent with the findings of a field survey conducted. The results of this study provide guidance and reference for future work on the technical assessment of mining-related damage in mountainous areas. However, this study explored building damage solely in terms of slope stability, without consideration of other geological and natural factors. Therefore, further research on the technical assessment of mining-related damage in mountainous areas and in areas with complex geological conditions is needed.

### Conclusions

This study investigated surface cracks and building damage associated with mining activity in a mountainous area of China. Based on mining subsidence theory and slope stability analysis, it was determined that increasing slope instability (reflected in a decrease of the slope safety factor), which was attributable to the mining activity, was the principal reason for the damage caused to the buildings in the study area. On the other hand, the actual surface displacements obtained with InSAR technique also verified the existence of the slope slip phenomenon. The analysis results show that mining-induced surface deformation mining would not directly cause damage to buildings, but the occurrence of surface cracks due to underground mining could lead to slope instability, resulting in building damage. This finding established that the effects of mining activity could be transmitted beyond the boundary of influence, determined by conventional subsidence models. The novelty of this study is investigation of surface cracks and building damage associated with mining activity in a mountainous area of China based on mining subsidence theory and slope stability analysis, instead of the basic theory of mining subsidence (which cannot adequately explain the degree of damage to ground structures). The research results not only provide a reference for mining face design (especially in mountainous areas), but they can also facilitate early warning of building damage.

## Supporting information

S1 Appendix URL of Sentinel-1A and POD precise orbit ephemerides. (ZIP)

S2 Appendix Information of cross section. (ZIP)
